# *In Vitro* CRISPR-Cas12a-Based Detection of Cancer-Associated *TP53* Hotspot Mutations Beyond the crRNA Seed Region

**DOI:** 10.1089/crispr.2022.0077

**Published:** 2023-04-13

**Authors:** Kavish A.V. Kohabir, Lars O. Nooi, Arjen Brink, Ruud H. Brakenhoff, Erik A. Sistermans, Rob M.F. Wolthuis

**Affiliations:** ^1^ Department of Human Genetics, Amsterdam UMC location Vrije Universiteit Amsterdam, Amsterdam, The Netherlands.; ^2^ Embryonic & Fetal Development, Amsterdam Reproduction & Development, Amsterdam, The Netherlands.; ^3^ Imaging and Biomarkers, Cancer Center Amsterdam, Amsterdam, The Netherlands.; ^4^ Cancer Biology and Immunology, Cancer Center Amsterdam, Amsterdam, The Netherlands.; ^5^ Department of Otolaryngology/Head and Neck Surgery, Amsterdam UMC location Vrije Universiteit Amsterdam, Amsterdam, The Netherlands.

## Abstract

Cost-effective and time-efficient detection of oncogenic mutations supports improved presymptomatic cancer diagnostics and post-treatment disease monitoring. Clustered regularly interspaced short palindromic repeats (CRISPR)-Cas12a is an RNA-guided endonuclease that, upon protospacer adjacent motif (PAM)-dependent recognition of target DNA *in cis*, exhibits indiscriminate ssDNase activity *in trans*, which can be harnessed for diagnostics. *TP53,* one of the most frequently mutated tumor suppressor genes in cancer, displays recurring point mutations at so-called “hotspots.” In this study, we optimized Cas12a-based assay conditions for *in vitro* detection of six *TP53* hotspot mutations at the codon for p.R273, located outside the Cas12a seed region, and evaluated the specificities of four commercial Cas12a variants. We found that nonengineered LbCas12a significantly outperformed the other tested nucleases specifically in distinguishing mutant p.R273 codons in synthetic DNA, mock cell-free DNA, and tissue biopsies, despite the suboptimal PAM-distal positioning of the corresponding mutations. Future clinical Cas12a-based applications may include point-of-care tumor analysis, cost-effective mutation screening, and improved monitoring of individual cancer patients.

## Introduction

Early, or even presymptomatic, diagnosis of cancer is correlated with more effective treatment and improved prognosis.^1^ Emerging advances in minimally invasive liquid biopsies demonstrate the potency of cell-free DNA (cfDNA) as a biomarker for the detection of genetic aberrations.^2^ These offer promising opportunities for early cancer screening and detection, or for post-treatment monitoring of potential relapse and minimal residual disease. However, many of the standardized detection technologies are based on next-generation sequencing, which is not always accessible and can be costly and laborious. Furthermore, continuously changing sequencing kits and platforms hamper a standardized diagnostic setting.

This drives the quest for more affordable, reliable, and point-of-care (PoC)-suitable alternatives to be used in routine testing and screening of pathological mutations. More than 50% of human cancers bear mutations in *TP53*, the gene coding for the transcription factor p53.^3^ These *TP53* mutations may dysregulate the native tumor suppressing characteristics of p53, whereas contributing to gain of oncogenic features. In particular mutations that impact the p53 DNA-binding domains are found most frequently, and are concentrated at “hotspot” positions in the coding sequence.^3,4^ Two of the most abundant pathogenic *TP53* mutations affect the codon for amino acid residue 273 of the protein (p.R273).

RNA-guided endonucleases associated with clustered regularly interspaced short palindromic repeats (CRISPR) loci found in bacterial adaptive immune systems have been successfully adapted for genome manipulation, including the introduction of insertions, deletions, base substitutions, and expression modulation.^5–7^ In particular class 2 systems, such as CRISPR-Cas9, have shown to be interesting candidates for these purposes, as the catalytic capacity is attributed to a single effector protein.^8^ Recently, Cas12a (previously named Cpf1) was characterized as a single effector that matures its own CRISPR RNA (crRNA), prefers targets with 5′ proximal T-rich protospacer adjacent motif (PAM) sites and cleaves DNA through other catalytic domains, leading to its classification as a distinct type of Class 2 effector.^9^ Cas12a was found to poorly tolerate mismatches between the PAM-proximal pentanucleotide region of the protospacer and the hybridizing part of the crRNA, which is defined as the seed region.^9,10^

Chen et al revealed that upon PAM-dependent target recognition and subsequent cleavage *in cis*, Cas12a proceeds to indiscriminately digest surrounding DNA, referred to as “collateral cleavage” or “*trans*-cleavage.”^11^ By including a quenched fluorescent single-stranded DNA (ssDNA) probe in their reactions, the same authors adopted this nuclease property to extend the use of CRISPR for diagnostics, naming their method “DNA endonuclease-targeted CRISPR trans reporter” (DETECTR).^11^ Discovery of collateral activity in other CRISPR families further expanded the CRISPR toolbox to double-stranded DNA (dsDNA), ssDNA, and RNA detection by methods such as SHERLOCK, HOLMES, CDetection, and Cas14-DETECTR.^12–16^ CRISPR-based diagnostics (CRISPRDx) have also been adapted to detect proteins, metabolites, antibiotics, and even minerals.^17–20^ CRISPRDx are cheap, simple, quick, accessible, PoC-deployable, and yet offer reprogrammable sequence-based specificity as nucleases are targeted toward sequences of interest.

Similar to all CRISPR-based technologies, Cas12a-based diagnostics rely on nuclease fidelity and capacity to discriminate between on- and off-targets. With substantial mismatch tolerance outside of the seed region, Cas12a can be used most effectively for detection of mutations that occur within five nucleotides downstream of the PAM.^11^ In combination with a stringent tetranucleotide T-rich PAM, this narrows down the scope of Cas12a-targetable clinically relevant mutations.

In this study, we demonstrate and compare improved Cas12a-based detection of nonseed region single nucleotide hotspot missense mutations in the *TP53* codon p.R273. We first explored the diagnostic assay setup and optimal approach for comparative quantification. Next, we assessed four different commercially available Cas12a orthologues on their ability to distinguish between point mutations in synthetic *TP53* sequences. We demonstrate that, in combination with optimized amplification settings, we can detect relevant *TP53* p.R273 mutations in mock cfDNA samples derived from culture medium used for mutant p53 cell lines.

Finally, we show that our setup is effective to pick up the respective oncogenic mutant codon in tumor biopsies from cancer patients. Our results indicate that CRISPR-Cas12a tumor genomics can be used to rapidly detect and monitor targeted cancer-related mutations in research and preclinical studies. Future applications could include, but are not limited to, (pre)cancer screening, tumor genome analysis at the point of clinical care (e.g., at the surgery room), cost-effective diagnostics and monitoring of individual cancer patients, or individuals genetically predisposed to cancer.

## Materials and Methods

### Mammalian and bacterial cell cultures

Commercial mammalian cell lines and corresponding media used for this study are listed in Table 1. Cells were cultured at 37°C and 5% CO_2_ in a stationary Heracell 240i incubator (Thermo Fisher Scientific) until confluent. Medium was aspirated and spun down at 500 *g*, followed by transferring the supernatant to a separate vial, which was kept at −20°C until further use. Plasmid cloning and propagation was done in *Escherichia coli* subcloning efficiency DH5α competent cells (Invitrogen™, Thermo Fisher Scientific).

**Table 1.** Overview of human cell lines used in this study

***Name***	***Full cell line name***	***Tissue of origin***	***TP53 gene mutation***	***p53 Protein mutation***	***Culture medium***
WM9	WM -9	Melanoma	Wild type	Wild type	IMDM +10% FBS
C33A	C-33 A	Cervical carcinoma	c.817C>T	p.R273C	DMEM +10% FBS
H1975	NCI-H1975	Lung adenocarcinoma	c.818G>A	p.R273H	DMEM +8% FBS
VU1131	VU-SCC-1131	Floor of mouth squamous cell carcinoma	c.818G>T	p.R273L	DMEM +8% FBS
HCC38	HCC38	Breast ductal carcinoma	c.818G>T	p.R273L	RPMI 1640 + 10% FBS

*All mutations are homozygous*.

DMEM, Dulbecco's modified Eagle's medium; FBS, fetal bovine serum; IMDM, Iscove's modified Dulbecco's medium.

Bacteria were cultured in liquid Luria-Bertani (LB) medium complemented with 100 mg/L ampicillin, prepared from Difco™ LB broth, Lennox (Becton, Dickinson and Company), or Difco Agar Noble (Becton, Dickinson and Company) in case of solid medium. Solid media were complemented with 500 nM IPTG and 100 mg/L X-gal for blue-white selection. Bacterial cultures were incubated at 37°C, in a Unitron^®^ plus incubator shaker at 200 rpm (Infors HT) or a Heraeus stationary incubator (Thermo Fisher Scientific).

### Patients and tissue specimen

Biopsy specimen obtained from previous work were reused in this study.^21^ In brief, biopsies were obtained from head and neck squamous cell carcinoma (HNSCC) patients during preoperative examination under general anesthesia. Written informed consent was obtained of patients enrolled in the study. The protocol was approved by the Institutional Review Board (2008/71). Tissue was immediately snap-frozen and stored in liquid nitrogen. Mutations were sequenced by Sanger sequencing of *TP53* exon 4–10.^21^ For this study, one tumor biopsy with a mutation in codon p.R273 and one tumor biopsy with wild-type sequence in that codon were selected for DNA isolation.

For each selected tumor biopsy, a paired deep connective/muscle tissue biopsy sample from the respective patient was taken along as a control. Biopsy selection was done by a collaborator and labels were encrypted until retrospective validation to confirm the mutational status. For this study, Sanger sequencing was repeated on *TP53* exon 8 polymerase chain reaction (PCR) amplicons from the selected tissue samples, using the Mix2Seq kit (Eurofins Scientific), in combination with primers KD050 and KD051 (Supplementary Table S1).

### DNA isolation from culture media and tissue biopsies

DNA was isolated from thawed mammalian cell culture medium supernatant through automated nucleic acid extraction using the QIAsymphony^®^ (Qiagen). Fifty microliters elutes from the same culture medium were pooled and subsequently quantified using the Qubit™ dsDNA HS kit (Invitrogen, Thermo Fisher Scientific) on a Qubit 4 Fluorometer (Invitrogen, Thermo Fisher Scientific). Isolates were stored at −20°C. Mock cfDNA samples derived from cell culture were separated on a 1.5% UltraPure™ agarose gel (Invitrogen, Thermo Fisher Scientific) with 0.05% (v/v) EtBr and visualized with a Gel Doc XR+ imager (Bio-Rad Laboratories, Inc.) to confirm presence of expected nucleosomal fragment sizes.

For the tumor fraction simulation assay, mock cfDNA from VU1131 was diluted with mock cfDNA obtained from a *TP53* wild-type cell line (WM9). Tissue biopsies were subjected to macrodissection before DNA isolation, ensuring that neoplastic cellularity was at least 20%. Subsequent DNA isolation was done using the Purelink™ Genomic DNA Mini Kit (Cat. No. K182001; Invitrogen, Thermo Fisher Scientific). Nucleic acid concentration and purity were assessed using a NanoDrop One microvolume Spectrophotometer (Thermo Fisher Scientific).

### Synthetic activator constructs

The ssDNA and single-stranded RNA (ssRNA) oligonucleotides used for this study were obtained from Integrated DNA Technologies, Inc. (IDT) and are listed in Supplementary Table S1. A panel of short dsDNA activator oligonucleotides was constructed by 1:1 mixing complementary ssDNA oligonucleotides, heating to 95°C for 3 min and cooling down to ambient temperature to anneal. The resulting panel of short dsDNA activators harbored different p.R273 codons in the middle and was used directly in CRISPR detection reactions and as insert for KpnI/EcorI cloning into pUC19 multiple cloning sites.

Constructs were heat-shock transformed to *E. coli*, grown on solid media overnight and checked by blue-white screening, PCR, and Sanger sequencing using the Mix2Seq kit (Eurofins Scientific), in combination with primers KD050 and KD051. Verified clones were used to inoculate overnight liquid cultures from which plasmids were isolated using Pure Yield™ Plasmid Midiprep system (Promega Corporation) and quantified using a Nano Drop One microvolume Spectrophotometer (Thermo Fisher Scientific).

### Plasmid *cis*-cleavage assays

AsCas12a Ultra and LbCas12a Ultra were purchased from IDT. These proteins are rationally or evolutionarily engineered by the manufacturer for optimized for genome editing. AsCas12a V3 and EnGen^*®*^ LbCas12a are wild-type Cas12a orthologues, purchased at IDT and New England Biolabs, respectively. Cas12a *cis*-cleavage assays were done using the pUC19-derived plasmid library (Supplementary Fig. S1). Ten nanomolars Cas12a was used to cut excess plasmid DNA in 90 min at 37°C in 1× CutSmart^®^ buffer (New England Biolabs). crRNAs were used in a ratio of Cas12a:crRNA equal to 1:1.25. Reaction products were separated on a 1.5% UltraPure agarose gel (Invitrogen, Thermo Fisher Scientific) with 0.05% (v/v) EtBr and visualized with a Gel Doc XR+ imager (Bio-Rad Laboratories, Inc.) to assess plasmid topology and Cas12a *cis*-activity.

### *TP53* exon 8 amplification

PCR amplification of *TP53* loci containing the p.R273 codon, or mutants thereof, was done using primers KD046 and KD047, which enclose the first 73 base pairs of exon 8. Using Q5^®^ Hot Start High-Fidelity DNA Polymerase (New England Biolabs), a final template concentration of 0.2 ng/μL and an annealing temperature of 68°C for an indicated amount of cycles, 73 bp amplicons were synthesized with codons of interest in the middle. Reaction products were visualized on a 1.5% UltraPure agarose gel (Invitrogen, Thermo Fisher Scientific) with 0.05% (v/v) EtBr and visualized with a Gel Doc XR+ imager (Bio-Rad Laboratories, Inc.).

### Fluorescent reporter *trans*-cleavage assays

*Trans*-cleavage reactions were carried out in 20 μL final volume in a Low Volume 384-well Black Flat Bottom Polystyrene NBS Microplate (Corning^®^). Reactions contained 1 × CutSmart buffer (New England Biolabs) complemented with 100 nM ssDNA probe labeled with a Iowa Black^®^-quenched 6-carboxyfluorescein (6-FAM) dye, as described previously.^11^ Assays were performed using a 1:1.25 ratio of Cas12a:crRNA. Reactions were initiated by adding 50 bp synthetic DNA activators to a final concentration of 50 pM, or by adding 1.5 μL of a raw PCR reaction mixture. For the combinatorial assays, omitted components were replaced by water to reach 20 μL final volume. Reactions were incubated for an indicated duration at 37°C, while monitoring fluorescence (λ_ex_ = 485 nm, λ_em_ = 535 nm) every 5 min in an Infinite^®^ 200 Pro M Plex plate reader (Tecan Group Ltd.).

Alternatively, measurements were done using the LightCycler^®^ 480 (Roche Diagnostics), equipped with filter sets (λ_ex_ = 465 nm, λ_em_ = 510 nm) for fluorescence measurements. Reactions were carried out in triplicate, unless otherwise stated. Each replicate series contained reactions without activators to monitor subtractable background signal. Mean background-subtracted fluorescence values and corresponding standard deviations were plotted for fluorescence curves. Slope heat maps were derived by linear regression on each replicate series over the indicated time window and normalizing the mean of the resulting slopes. Slope normalization was done respective to the highest value for every tested crRNA, yielding a percentage. Two-way analysis of variance tests were done to assess whether obtained mean slope values from triplicate experiments were significantly different.

## Results

### Target selection and assay design

**FIG. 1.**
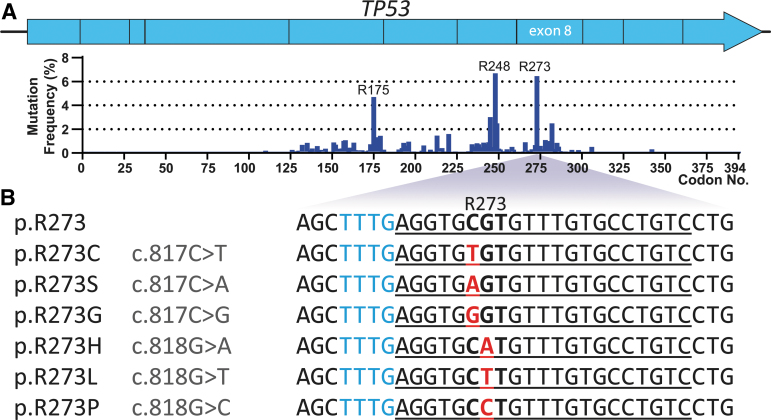
Overview of p.R273 mutations in *TP53* used to design crRNAs for Cas12a-based detection. **(A)** Mutation frequency distribution across the *TP53* gene indicate R273 as one of the most frequently mutated codons. Data were obtained from *The TP53 Database (R20, July 2019)*.^4^ **(B)** Relevant R273 missense mutations (red) are found downstream of a Cas12a-compatible PAM-site (blue), at position 6/7 of the target sequence (underlined). Denotations for missense point mutations and resulting protein mutations are listed next to the corresponding highlighted sequences. crRNA, CRISPR RNA; PAM, protospacer adjacent motif.

Out of all *TP53* codons, p.R273 is the second-most frequently mutated codons in *TP53*, with a frequency of ∼7% (Fig. 1A). p.R273C (c.817C>T) and p.R273H (c.818G>A) are among the top five most common p53 hotspot mutations, together found in roughly 7% of widespread human cancers.^3^ Less frequent p.R273 mutations include p.R273L (c.818G>T), p.R273S (c.817C>A), p.R273G (c.817C>G), and p.R273P (c.818G>C). All of these mutant codons are preceded by a TTTG tetranucleotide sequence that matches the PAM preferences of Cas12a, and is located 6 nucleotides upstream of the mutated codon. Cas12a crRNAs were designed to target p.R273C, p.R273H, p.R273L, p.R273S, p.R273G, and p.R273P mutant codons in exon 8 of *TP53* (Fig. 1B). The full Cas12a-crRNA panel was tested for its capability to recognize and *cis*-cleave plasmids with target site inserts containing the corresponding mutations (Supplementary Fig. S1).

**FIG. 2.**
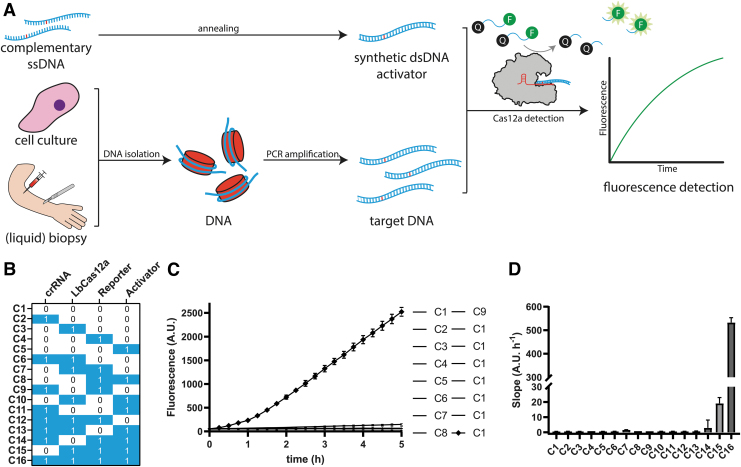
Schematic diagram of Cas12-based detection of *TP53* p.R273 point mutants. **(A)** Short complementary ssDNA oligonucleotides are annealed to form synthetic dsDNA activators that harbor wild-type or mutant *TP53* sequences. Cell-free genetic material is isolated from cell culture media or biopsies and followed by PCR amplification. Obtained amplicons or synthetic dsDNA activators are used to complement the incubated Cas12a reaction containing a quenched (Q) fluorescent (F) probe. As a consequence of target DNA recognition *in cis*, the probe is *trans*-cleaved and an increasing fluorescence signal is monitored periodically over time. **(B)** Overview of the presence (1) or absence (0) of reagents in reactions C1–C16 of the combinatorial assay. **(C)** Mean fluorescence values of three replicate combinatorial assays, plotted against time, measured on a Tecan infinite^®^ 200 Pro plate reader. C16 is the only reaction yielding significant fluorescence above background. Error bars display the standard error of the mean. **(D)** Mean curve slopes from three replicate combinatorial assays measured on a Tecan Infinite 200 Pro plate reader. Error bars display standard error of the mean. dsDNA, double-stranded DNA; PCR, polymerase chain reaction; ssDNA, single-stranded DNA.

To make use of the collateral activity for Cas12a-based diagnostics, reactions were complemented with a quenched fluorescent ssDNA probe and a target DNA sequence acting as activator, combined in a reaction buffer. Upon *in cis* PAM- and crRNA-dependent recognition of target DNA, hereafter referred to as activators, Cas12a is expected to cleave the probe *in trans*, resulting in fluorescence signal increase over time (Fig. 2A). To ensure that the detection method would exclusively detect signal in the presence of all required components, we performed a combinatorial assay, in which all combinations of components have been monitored for fluorescence signal over time (Fig. 2B).

In this study, we used small synthetic dsDNA oligonucleotides harboring the relevant p.R273 mutations as activators, for a time course of 5 h in a temperature-controlled microplate reader. Out of all tested possible reaction compositions, the only reaction significantly increasing in fluorescence yield contained all required components for Cas12a-based DNA detection through collateral activity (Fig. 2C). The slope of that resulting curve is significantly steeper than that of the other reactions, including the ones lacking target DNA, suggesting that curve slopes could be an appropriate translated measure to evaluate presence of target DNA in detection reactions (Fig. 2D). We repeated this on a LightCycler 480 to demonstrate feasibility of this CRISPRDx assay on quantitative PCR machinery (Supplementary Fig. S2).

### Mismatch tolerance analysis

The point mutations of interest are positioned at PAM +6 or PAM +7 (Fig. 1B), which is just outside of the 5-nucleotide PAM-proximal region that hybridizes with the crRNA seed region, in which Cas12a maintains very low tolerance to mismatches.^9^ This may pose challenges for the nuclease to distinguish between different mutations from the panel, as well as between the panel and the wild-type sequences. Therefore, it is important to select an effector nuclease capable of discriminating mutations at these positions outside of the seed region.

Although there is a plethora of characterized Cas12a orthologues, only a limited selection is commercially available, including Cas12a from *Lachnospiraceae bacterium* (LbCas12a)*, Acidaminococcus* sp. (AsCas12a), and engineered derivatives for improved activity and broader PAM recognition.^22–25^ Engineered variants marked with the suffix *Ultra* have been optimized for genome editing in cells, which may not reflect the cell-free environment of our detection reactions. We tested and compared single-nucleotide specificities of all four Cas12a variants that were commercially available at the time of this study: AsCas12a Ultra, LbCas12a Ultra, AsCas12a V3, and EnGen LbCas12a.

**FIG. 3.**
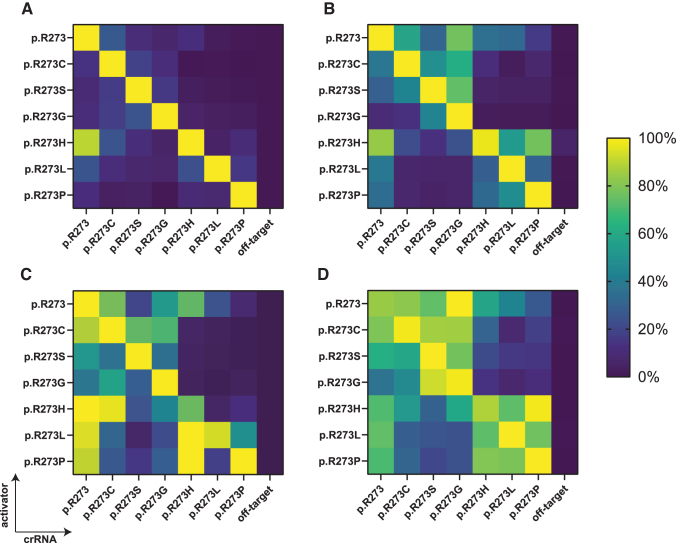
Cas12a mismatch tolerance analysis for mutant *TP53* p.R273 detection. A panel of crRNAs and short dsDNA activators harboring p.R273 mutations was shuffled to assess mismatch tolerance of **(A)** 20 nM EnGen^®^ LbCas12a, **(B)** 4 nM AsCas12a V3, **(C)** 4 nM LbCas12a Ultra, and **(D)** 4 nM AsCas12a Ultra at the relevant distance from the PAM. Curve slopes were calculated from background-subtracted data and were percentually normalized to the steepest slope value for a given activator. A crRNA with a nontargeting synthetic spacer that does not match any of the activators (“off-target”) was used as negative control. All figures represent average values of three independently performed experiments. Fluorescence curves and non-normalized slopes are shown in Supplementary Figures S4 and S5.

The Ultra enzymes are evolutionarily engineered for improved genome editing efficiency, whereas the remaining two variants are wild-type Cas12a enzymes.^22,24^ We found that in a cell-free environment, engineered LbCas12a and both AsCas12a variants are more active when compared with wild-type LbCas12a, requiring less enzyme to achieve the same kinetics (Supplementary Fig. S3). We assessed each candidate further in a cell-free environment on its tolerance to internal mismatches within the designed p53 panel of activators and crRNAs, by comparing the fluorescence curve slopes of the individual reactions with each other. For each tested enzyme, the resulting ratios were expressed as percentages and plotted in heat maps (Fig. 3).

Our results reveal that the most specific discrimination between on- and off-targets was found using nonengineered LbCas12a, where all mutations were specifically recognized except for the p.R273H activator, which non-specifically activated collateral activity in the presence of a crRNA targeting the wild-type codon sequence (Fig. 3A). This was not the case for the reverse situation, since the crRNA directed to detect codon p.R273H showed no off-target activity on wild-type sequence activator. Changing the enzyme concentration did not disturb this specificity profile (Supplementary Fig. S6A).

We attempted to reduce off-target activity by introducing an adjacent mismatching base in the crRNA, in a similar manner as was done by Huang and colleagues, but found that the intended on-target activity was severely impacted as well (Supplementary Fig. S6D).^26^ AsCas12a V3, the other commercially available wild-type Cas12a variant, exhibited moderate capacity to distinguish between full matches and single-nucleotide mismatches, tolerating a moderate level of off-target activity (Fig. 3B). LbCas12a Ultra (Fig. 3C) and AsCas12a Ultra (Fig. 3D) strongly tolerated target mismatches within the designed panel. For some activator-crRNA combinations, the Ultra enzyme off-target activity was even stronger than that of the activator-crRNA set without mismatches.

Based on the observed mismatch tolerances, we decided to proceed with wild-type LbCas12a to further assess detection of the p.R273 mutant panel in the presence of background genomic DNA, reflecting the nucleic acid makeup of liquid biopsies in a better way. It has been observed before that cells in culture shed fragmented pieces of genomic DNA with oligonucleosomal size distributions as found for cfDNA in plasma.^27,28^ Therefore, we aimed to mimic liquid biopsy cfDNA by extracting nucleic acids from medium used for culturing *TP53* mutant cell lines C33A, H1975, VU1131, and HCC38 (Table 1).

These cell lines homozygously encode p.R273C, p.R273H, pR273L, and p.R273L, respectively, the clinically most prevalent mutant p.R273 variants. In addition, we used cell line WM9 that contains only wild-type *TP53* alleles. We isolated nucleic acids from the medium used for culturing these cancer cell lines, in a manner identical to isolation from blood plasma. The obtained nucleic acids indeed reflected patterns of *n*-mers of nucleosomal fragment sizes (Supplementary Fig. S7) that are concordant with existing literature on plasma cfDNA fragmentomics.^29^

**FIG. 4.**
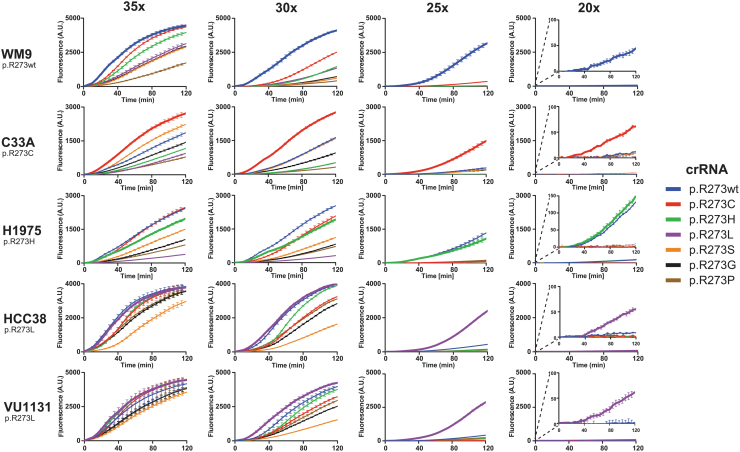
Excessive amplification by PCR promotes off-target activity on cell-line derived genetic material. DNA isolated from growth medium used for culturing WM9, C33A, H1975, HCC38, and VU1131 was subjected to PCR with either 35, 30, 25, or 20 cycling steps. Cell lines and corresponding homozygous p53 mutations are displayed on the left. The PCR mixtures served as activators in crRNA-specific LbCas12a diagnostic reactions for 2 h in an incubating plate reader. Plotted curves represent mean background-subtracted data from three replicate experiments. Error bars display standard deviation of the mean. Reaction curves representing crRNAs that match R273 codons of a respective cell line are drawn thicker, indicating reactions that are expected to yield signal due to on-target activity.

Cas12a-based diagnostics may require target amplification to overcome the detection limit of the enzyme. Next, we optimized the number of PCR amplification cycles required for Cas12a to detect mutant *TP53*. If the detection is specific, fluorescence signal should only increase in reactions that contain a crRNA that matches the mutation found in the respective cell line. At 30 or 35 cycles, excessive target amplification promoted all reactions to yield rapid fluorescence signal increase, irrespective of the supplied crRNA (Fig. 4). In contrast, at 20 cycles, fluorescence increase is only marginal. However, this marginal signal increase appears to be specific and originates only from reactions with crRNAs and corresponding activating cell line mutations. At 25 PCR cycles, we find an optimal trade-off between both significant and specific signal increase.

**FIG. 5.**
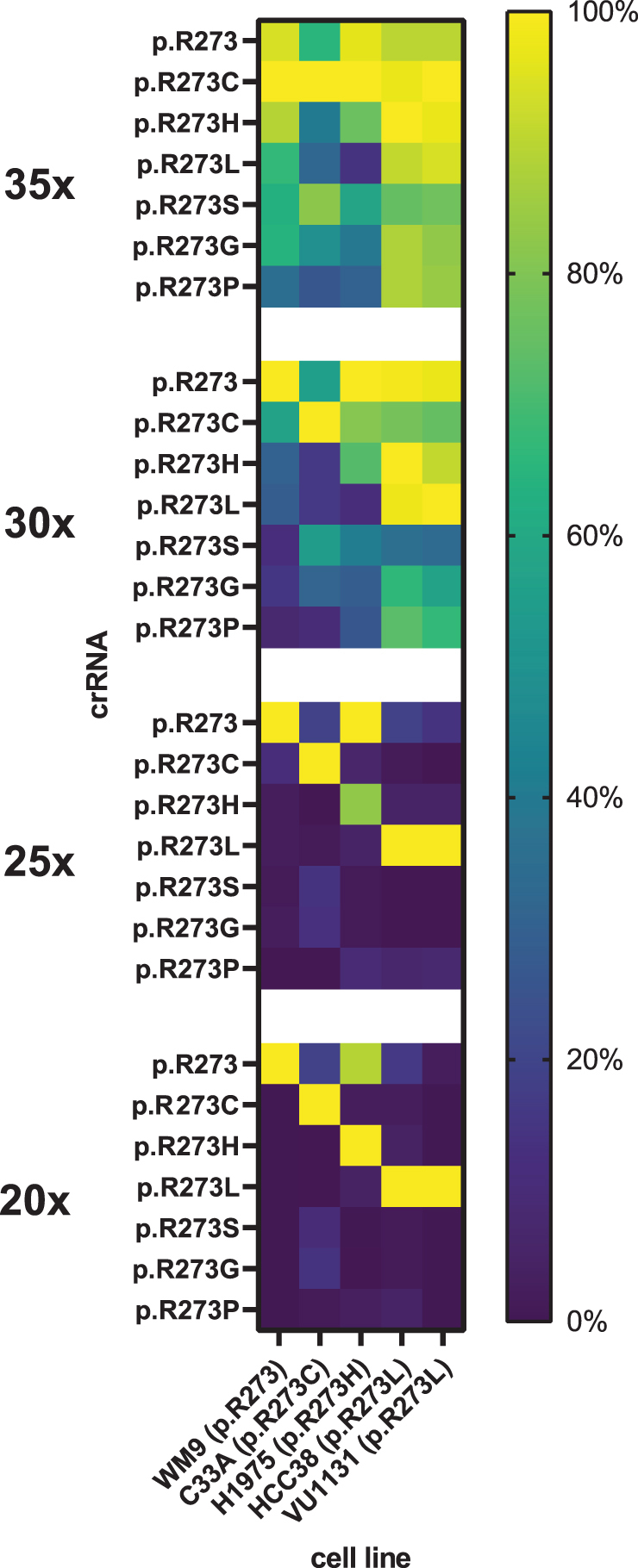
Excessive target amplification by PCR stimulates off-target activity. Heat maps display normalized mean slope values of 2-h LbCas12a reactions with a crRNA (rows) and PCR amplification (35×, 30×, 25×, or 20× cycling steps) on tumor cell lines (columns). Normalization was done against highest value per tested cell line, for each of the tested amount of PCR cycles. Data represent triplicate experiments.

Comparing the curve slopes confirms that excessive amplification stimulates off-target activity relatively more than it does for on-target activity (Fig. 5). Furthermore, we observed the same persistent off-target activity as before (Fig. 3A), where the crRNA recognizing the wild-type codon also recognizes the p.R273H codon. The data from cell line WM9 confirm that, at up to 25 amplification cycles, the crRNA for detecting p.R273H remains specific, and does not recognize the wild-type codon. This suggests that our designed crRNA panel can distinguish between all possible single-nucleotide missense hotspot mutations in *TP53* codon p.R273. Our dual setup of PCR and wild-type LbCas12a-based detection thus allows for rapid identification of presence of these hotspot mutations in the context of highly fragmented genomic DNA.

**FIG. 6.**
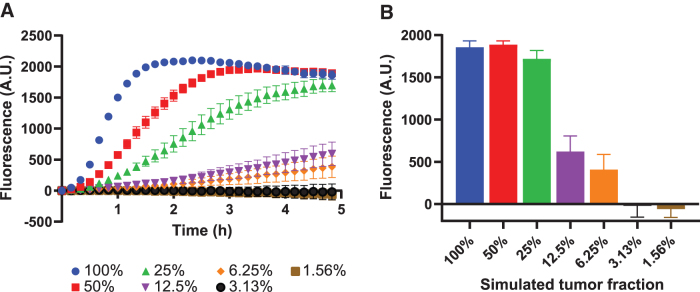
LbCas12a detects simulated tumor fractions down to at least 6%. **(A)** Tumor fraction simulation assay data over time. A factorial dilution series of VU1131 cfDNA mimic in WM9 cfDNA mimic was subjected to 25 PCR cycling steps, followed by CRISPRDx for 5 h. Curves display mean background fluorescence values of a triplicate experiment. Background subtraction was done using mean fluorescence values of a 0% tumor fraction triplicate. Error bars indicate standard errors of the mean. **(B)** Bars represent end point measurements of three replicate experiments. Error bars display standard deviation of the mean. cfDNA, cell-free DNA; CRISPR, clustered regularly interspaced short palindromic repeats; CRISPRDx, CRISPR-based diagnostics.

The used cfDNA mimic was derived from mutant cell lines that are homozygous for the respective mutations. This implies that all *TP53* copies in the sample harbor the mutation, thus resembling a sample with a 100% tumor fraction, which is above the clinically relevant range of fractions of <10%.^30^ To determine to which degree our assay can specifically detect the mutated allele in the presence of wild-type alleles, we diluted mutant mock cfDNA in mock cfDNA derived from a wild-type *TP53* cell line, and subjected this dilution series to CRISPRDx (Fig. 6A). This assay demonstrates that, using our setup, a lower limit of detection around a simulated tumor fraction of 3–6% was achievable (Fig. 6B).

### Testing biopsy samples

More than 70% of reported HNSCC cases have an inactivating mutation in *TP53*, most of which are hotspot mutations.^31^ As a demonstration of principle, we proceeded to test extracts from frozen primary HNSCC biopsies from two patients, by means of a blinded experiment, without prior knowledge of the mutational status of codon p.R273. Each tumor biopsy was paired with healthy muscle tissue biopsy taken from the same patient, distantly from the HNSCC sites, and should harbor only the wild-type *TP53* codon. *TP53* exon 8 loci were amplified through 25 cycles of PCR amplification, followed by a 2-h detection assay using wild-type LbCas12a. After background subtraction and regression, we found that the wild-type p.R273 allele caused highest probe cleavage activity in all tested biopsy samples (Supplementary Fig. S8).

**FIG. 7.**
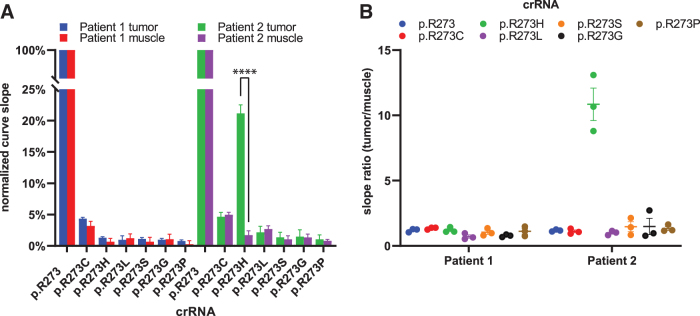
LbCas12a-based detection of a *TP53* hotspot mutation in HNSCC tissue biopsy. **(A)** Mean background subtracted fluorescence curve slopes normalized as percentages of the highest slope value for each tested biopsy sample. In paired biopsies of patient 2, Cas12a detects a significantly higher amount of p.R273H codons in the tumor biopsy with ****p < 0.0001. Bars represent mean values, error bars standard deviation of the mean, from triplicate experiments. **(B)** Slope ratios of paired biopsies (tumor/muscle) for the tested crRNA panel demonstrate that LbCas12a identified patient 2, and not patient 1, with a hotspot mutation in p.R273. Error bars display standard deviation of the mean, calculated from triplicate experiments. Detection was done on PCR-amplified (25 cycles) biopsy DNA isolate. Original fluorescence curves are shown in Supplementary Figure S8. HNSCC, head and neck squamous cell carcinoma.

Curve slopes were normalized to the highest slope value for each biopsy data set, fixing the p.R273wt crRNAs at 100% (Fig. 7A). The assay did not pick up differences between muscle and HNSCC biopsies for patient 1, suggesting that the makeup of p.R723 codons is predominantly wild type in both tissues. In tumor tissue of patient 2, LbCas12a picks up presence of p.R273H codons, whereas it explicitly does not do this in muscle tissue from the same patient. This significant (*p* < 0.0001) difference between the two tissues was only found for reactions detecting the p.R273H codon, other reactions yielded comparable signal between tumor and muscle tissue.

By taking the ratio (tumor/muscle) of the background-subtracted slope values, we visualized the more than 10-fold steeper signal increase when detecting p.R273H in HNSCC tissue of patient 2 compared the paired muscle tissue (Fig. 7B). None of the other crRNAs in the panel detected a mutation different from the background signal found in muscle tissue, approaching a slope ratio of 1. These results suggest that LbCas12a identified a *TP53* p.R273H hotspot mutation in the tumor of HNSCC patient 2. Retrospective blinded validation with the available sequencing data of the tissue specimens confirmed this (Supplementary Fig. S9).

## Discussion

In this study, we optimized Cas12a-based assay conditions for *in vitro* detection of six *TP53* hotspot mutations in the codon for p.R273, located outside the Cas12a seed region, in synthetic DNA, mock cfDNA, and tissue biopsies. We showed that our diagnostic Cas12a assay setup requires all reaction components to yield significant fluorescence signal, and that comparing fluorescence curve slopes offers a measure for quantitative comparisons. Out of the four tested commercially available Cas12a enzymes, the nonengineered LbCas12a variant performed with highest single-nucleotide fidelity on the designed p.R273 mutation panel. Using this effector, and optimized amplification conditions, we were able to resolve the *TP53* p.R273 codon compositions of multiple mutant p53 cell lines, despite the fact that these hotspot mutations are outside the pentanucleotide seed region following the 5′ PAM site. As a final proof of concept, we demonstrated Cas12a-based detection using the designed crRNA panel on HNSCC patient biopsies.

We tested all commercially available Cas12a variants at the time of this study. Our findings on intrinsic specificity differences between the tested wild-type Cas12a orthologues AsCas12a and LbCas12a are in line with previous findings.^32^ AsCas12a Ultra and LbCas12a Ultra are proprietary engineered variants, optimized for higher activity to improve the genome editing efficiency in cells. AsCas12a Ultra, for instance, is an evolutionarily engineered variant with mutations in the PAM- and crRNA-interacting domains, conferring higher activity, a more relaxed PAM preference and maintained on-target fidelity.^22^ These enzymes, however, have not been benchmarked for their fidelity in cfDNA detection assays. In this study, we demonstrated that, although activity of engineered variants is significantly higher in cell-free environments, specific detection of mutations at PAM +6 and PAM +7 was compromised, suggesting a trade-off between enzyme activity and specificity under these conditions.

Nonengineered LbCas12a significantly outperformed the other tested variants in terms of specificity, but did not yield 100% fidelity either. Consistently, for this wild-type LbCas12a, we found that presence of p.R273H codons activated the nuclease in both reactions with crRNAs detecting p.R273H and wild-type p.R273. Since we expect to detect wild-type *TP53* in clinical samples either way, we suggest that the crRNA directed at wild-type p.R273 could still be used as a positive control, since the all other *TP53* crRNAs in the panel are mutation specific. The position of the tolerated mismatch for this mutation is at PAM +7, furthest from the seed region of all tested combinations. Contrarily, presence of the wild-type p.R273 codon did not activate Cas12 with crRNA detecting the p.R273H codon.

These results suggest that, apart from the position of the mismatch, also the type of mismatched base pair influences the degree of mismatch tolerance in Cas12a assays. LbCas12a tolerated a dG·rU mismatch between the crRNA and the target strand, which are able to form a wobble base pair. Recent findings on structural Cas9 biology identified such noncanonical base pairing as a contributing factor to off-target activity.^33^ If this also holds for Cas12a, it may explain the observed LbCas12 off-target activity. Incorporating this in future assay design can anticipate false positive results and achieve higher testing accuracy. Use of other Cas12a orthologues or recently characterized type V CRISPR effectors such as Cas12b, Cas12c, Cas14, and CasΦ that make use of noncanonical PAM sites may offer more flexible mutation detection within the respective seed regions.^11,14,15,34,35^ Alternatively, studies showed that engineering the crRNA may also improve specificity.^36–38^

Cas12a has an amplification-free detection limit in the picomolar range.^11,39^ There are speculations that this is the case for most CRISPR detection effectors.^40^ Since nucleic acid levels in liquid biopsies are significantly lower than the picomolar range, there is need for a form of enrichment.^41^ We identified that increased PCR amplification triggers off-target activity. Presumably, the chance of tolerating mismatches outside the seed region increases with the amount of target-like DNA in the reaction.

Too little or no amplification, however, prevented detection by Cas12a in our assays, resulting in false negatives. We found an optimal equilibrium between sensitivity and specificity at 25 PCR cycles, where a moderate but significant signal was obtained. The assay could be further optimized by using other methods, such as isothermal amplification techniques (e.g., loop-mediated isothermal amplification [LAMP] or recombinase polymerase amplification [RPA]) to simplify sample preparation or even combine amplification with CRISPR detection into a one-pot reaction.^42–44^

This study made use of cell culture medium to extract cell-free fragmented oligonucleosomal DNA that reflects size patterns as found in plasma DNA.^29^ Such cfDNA mimics can be of good use in translational liquid biopsy research that requires cfDNA as input. As we demonstrated, these cfDNA mimics can be combined to simulate more complex allele compositions, which allowed us to approach different tumor fractions. Alternatively, cfDNA from a healthy individual may be spiked with a known amount of activator of interest to equilibrate the assay. DNA fragmentation poses hurdles for long-read sequencing, and requires deep short-read sequencing for enough coverage to resolve mutations on a single-nucleotide level.

We showed that it is possible for Cas12a enzymes to distinguish PAM +6 and PAM +7 mismatches on this fragmented DNA material, bringing CRISPRDx a step closer to application in liquid biopsies. Commonly, tissue biopsies are kept as formalin-fixed paraffin-embedded specimens, which is known to degrade DNA over time, hampering PCR- and sequencing-based methods.^45,46^ Our results demonstrate that activators can even be as small as 50 base pairs, and still be correctly identified by Cas12a, by-passing the limitations that established methods such as PCR and next-generation sequencing encounter due to DNA degradation.

As proof of concept, we demonstrated Cas12a-based identification of cancer-relevant mutations in blinded samples of tissue biopsies. We showed that under our optimized conditions, Cas12a can be purposed to detect mutations beyond the seed region with single-nucleotide fidelity. As controls, we used muscle biopsies that were taken distantly from the HNSCC sites, and were expected to reflect the wild-type *TP53* codon. The HNSCC tissue biopsies used in this study inevitably also contained non-neoplastic, or noncancerous, cells from the stroma due to the way biopsies are taken. Presence of these cells likely explains the detection of wild-type codons in all samples. In addition, patients may be heterozygously mutated, in which case half of the DNA targets from affected cells would still activate detection of the wild-type sequence.

## Conclusion

This study demonstrates an optimized Cas12a-based detection assay for detecting common oncogenic *TP53* hotspot mutations even though they reside beyond the seed region. We showed how selection of a proper Cas12a variant and amplification optimization impacts the specificity and efficacy when it comes to detecting single-nucleotide mutations at position PAM +6 and PAM +7. Furthermore, as proof of concept, we show that this method is effective in identifying the mutational status in tissue biopsies. The obtained results indicate that Cas12a holds promising diagnostic power, and may be further exploited for clinical and PoC detection of cancer.

## Supplementary Material

Supplemental data

Supplemental data

Supplemental data

Supplemental data

Supplemental data

Supplemental data

Supplemental data

Supplemental data

Supplemental data

Supplemental data

## References

[1] Neal RD, Tharmanathan P, France B, et al. Is increased time to diagnosis and treatment in symptomatic cancer associated with poorer outcomes? Systematic review. Br J Cancer 2015;112(Suppl 1):S92–S107; doi: 10.1038/bjc.2015.48 25734382 PMC4385982

[2] Wan JCM, Massie C, Garcia-Corbacho J, et al. Liquid biopsies come of age: Towards implementation of circulating tumour DNA. Nat Rev Cancer 2017;17(4):223–238; doi: 10.1038/nrc.2017.7 28233803

[3] Baugh EH, Ke H, Levine AJ, et al. Why are there hotspot mutations in the TP53 gene in human cancers? Cell Death Differ 2018;25(1):154–160; doi: 10.1038/cdd.2017.180 29099487 PMC5729536

[4] de Andrade KC, Lee EE, Tookmanian EM, et al. The TP53 database: Transition from the International Agency for Research on Cancer to the US National Cancer Institute. Cell Death Differ 2022;29(5):1071–1073; doi: 10.1038/s41418-022-00976-3 35352025 PMC9090805

[5] Adli M. The CRISPR tool kit for genome editing and beyond. Nat Commun 2018;9(1):1911; doi: 10.1038/s41467-018-04252-2 29765029 PMC5953931

[6] Komor AC, Badran AH, Liu DR. CRISPR-based technologies for the manipulation of eukaryotic genomes. Cell 2017;168(1–2):20–36; doi: 10.1016/j.cell.2016.10.044 27866654 PMC5235943

[7] Wang H, La Russa M, Qi LS. CRISPR/Cas9 in genome editing and beyond. Annu Rev Biochem 2016;85:227–264; doi: 10.1146/annurev-biochem-060815-014607 27145843 PMC13384723

[8] Shmakov S, Smargon A, Scott D, et al. Diversity and evolution of class 2 CRISPR-Cas systems. Nat Rev Microbiol 2017;15(3):169–182; doi: 10.1038/nrmicro.2016.184 28111461 PMC5851899

[9] Zetsche B, Gootenberg JS, Abudayyeh OO, et al. Cpf1 is a single RNA-guided endonuclease of a class 2 CRISPR-Cas system. Cell 2015;163(3):759–771; doi: 10.1016/j.cell.2015.09.038 26422227 PMC4638220

[10] Swarts DC, van der Oost J, Jinek M. Structural basis for guide RNA processing and seed-dependent DNA targeting by CRISPR-Cas12a. Mol Cell 2017;66(2):221.e4–233.e4; doi: 10.1016/j.molcel.2017.03.016 28431230 PMC6879319

[11] Chen JS, Ma E, Harrington LB, et al. CRISPR-Cas12a target binding unleashes indiscriminate single-stranded DNase activity. Science 2018;360(6387):436–439; doi: 10.1126/science.aar6245 29449511 PMC6628903

[12] Li SY, Cheng QX, Wang JM, et al. CRISPR-Cas12a-assisted nucleic acid detection. Cell Discov 2018;4:20; doi: 10.1038/s41421-018-0028-z 29707234 PMC5913299

[13] Gootenberg JS, Abudayyeh OO, Kellner MJ, et al. Multiplexed and portable nucleic acid detection platform with Cas13, Cas12a, and Csm6. Science 2018;360(6387):439–444; doi: 10.1126/science.aaq0179 29449508 PMC5961727

[14] Teng F, Guo L, Cui T, et al. CDetection: CRISPR-Cas12b-based DNA detection with sub-attomolar sensitivity and single-base specificity. Genome Biol 2019;20(1):132; doi: 10.1186/s13059-019-1742-z 31262344 PMC6604390

[15] Harrington LB, Burstein D, Chen JS, et al. Programmed DNA destruction by miniature CRISPR-Cas14 enzymes. Science 2018;362(6416):839–842; doi: 10.1126/science.aav4294 30337455 PMC6659742

[16] Kellner MJ, Koob JG, Gootenberg JS, et al. SHERLOCK: Nucleic acid detection with CRISPR nucleases. Nat Protoc 2019;14(10):2986–3012; doi: 10.1038/s41596-019-0210-2 31548639 PMC6956564

[17] Dai Y, Somoza RA, Wang L, et al. Exploring the trans-cleavage activity of CRISPR-Cas12a (cpf1) for the development of a universal electrochemical biosensor. Angew Chem Int Ed Engl 2019;58(48):17399–17405; doi: 10.1002/anie.201910772 31568601 PMC6938695

[18] Liang M, Li Z, Wang W, et al. A CRISPR-Cas12a-derived biosensing platform for the highly sensitive detection of diverse small molecules. Nat Commun 2019;10(1):3672; doi: 10.1038/s41467-019-11648-1 31413315 PMC6694116

[19] Mahas A, Wang Q, Marsic T, et al. Development of Cas12a-based cell-free small-molecule biosensors via allosteric regulation of CRISPR array expression. Anal Chem 2022;94(11):4617–4626; doi: 10.1021/acs.analchem.1c04332 35266687 PMC8943526

[20] Xiong Y, Zhang J, Yang Z, et al. Functional DNA regulated CRISPR-Cas12a sensors for point-of-care diagnostics of non-nucleic-acid targets. J Am Chem Soc 2020;142(1):207–213; doi: 10.1021/jacs.9b09211 31800219 PMC7174832

[21] van Houten VM, Tabor MP, van den Brekel MW, et al. Mutated p53 as a molecular marker for the diagnosis of head and neck cancer. J Pathol 2002;198(4):476–486; doi: 10.1002/path.1242 12434417

[22] Zhang L, Zuris JA, Viswanathan R, et al. AsCas12a ultra nuclease facilitates the rapid generation of therapeutic cell medicines. Nat Commun 2021;12(1):3908; doi: 10.1038/s41467-021-24017-8 34162850 PMC8222333

[23] Jacobsen T, Ttofali F, Liao C, et al. Characterization of Cas12a nucleases reveals diverse PAM profiles between closely-related orthologs. Nucleic Acids Res 2020;48(10):5624–5638; doi: 10.1093/nar/gkaa272 32329776 PMC7261169

[24] Chen P, Zhou J, Wan Y, et al. A Cas12a ortholog with stringent PAM recognition followed by low off-target editing rates for genome editing. Genome Biol 2020;21(1):78; doi: 10.1186/s13059-020-01989-2 32213191 PMC7093978

[25] Zetsche B, Abudayyeh OO, Gootenberg JS, et al. A survey of genome editing activity for 16 Cas12a orthologs. Keio J Med 2020;69(3):59–65; doi: 10.2302/kjm.2019-0009-OA 31723075 PMC7220826

[26] Huang X, Zhang F, Zhu K, et al. dsmCRISPR: Dual synthetic mismatches CRISPR/Cas12a-based detection of SARS-CoV-2 D614G mutation. Virus Res 2021;304:198530; doi: 10.1016/j.virusres.2021.198530 34363850 PMC8339451

[27] Bronkhorst AJ, Wentzel JF, Aucamp J, et al. Characterization of the cell-free DNA released by cultured cancer cells. Biochim Biophys Acta 2016;1863(1):157–165; doi: 10.1016/j.bbamcr.2015.10.022 26529550

[28] Bronkhorst AJ, Ungerer V, Holdenrieder S. Comparison of methods for the isolation of cell-free DNA from cell culture supernatant. Tumour Biol 2020;42(4):1010428320916314; doi: 10.1177/1010428320916314 32338581

[29] Kohabir K, Wolthuis R, Sistermans EA. Fragmentomic cfDNA patterns in noninvasive prenatal testing and beyond. J Biomed Transl Res 2021;7(1):38–47; doi: 10.14710/jbtr.v7i1.10229

[30] Bettegowda C, Sausen M, Leary RJ, et al. Detection of circulating tumor DNA in early- and late-stage human malignancies. Sci Transl Med 2014;6(224):224ra24; doi: 10.1126/scitranslmed.3007094 PMC4017867 24553385

[31] Cancer Genome Atlas Network. Comprehensive genomic characterization of head and neck squamous cell carcinomas. Nature 2015;517(7536):576–582; doi: 10.1038/nature14129 25631445 PMC4311405

[32] Murugan K, Seetharam AS, Severin AJ, et al. CRISPR-Cas12a has widespread off-target and dsDNA-nicking effects. J Biol Chem 2020;295(17):5538–5553; doi: 10.1074/jbc.RA120.012933 32161115 PMC7186167

[33] Pacesa M, Lin C-H, Cléry A, et al. Structural basis for Cas9 off-target activity. Cell 2022;185(22):4067.e21–4081.e21; doi: 10.1016/j.cell.2022.09.026 36306733 PMC10103147

[34] Yan WX, Hunnewell P, Alfonse LE, et al. Functionally diverse type V CRISPR-Cas systems. Science 2019;363(6422):88–91; doi: 10.1126/science.aav7271 30523077 PMC11258546

[35] Pausch P, Al-Shayeb B, Bisom-Rapp E, et al. CRISPR-CasPhi from huge phages is a hypercompact genome editor. Science 2020;369(6501):333–337; doi: 10.1126/science.abb1400 32675376 PMC8207990

[36] Jin X, Zhang L, Wang X, et al. Novel CRISPR/Cas12a-based genetic diagnostic approach for SLC26A4 mutation-related hereditary hearing loss. Eur J Med Genet 2022;65(2):104406; doi: 10.1016/j.ejmg.2021.104406 34968750

[37] Cunningham CH, Hennelly CM, Lin JT, et al. A novel CRISPR-based malaria diagnostic capable of Plasmodium detection, species differentiation, and drug-resistance genotyping. EBioMedicine 2021;68:103415; doi: 10.1016/j.ebiom.2021.103415 34139428 PMC8213918

[38] Krysler AR, Cromwell CR, Tu T, et al. Guide RNAs containing universal bases enable Cas9/Cas12a recognition of polymorphic sequences. Nat Commun 2022;13(1):1617; doi: 10.1038/s41467-022-29202-x 35338140 PMC8956631

[39] Lv H, Wang J, Zhang J, et al. Definition of CRISPR Cas12a T rans-cleavage units to facilitate CRISPR diagnostics. Front Microbiol 2021;12:766464; doi: 10.3389/fmicb.2021.766464 34912315 PMC8667580

[40] Huyke DA, Ramachandran A, Bashkirov VI, et al. Enzyme kinetics and detector sensitivity determine limits of detection of amplification-free CRISPR-Cas12 and CRISPR-Cas13 diagnostics. Anal Chem 2022;94(27):9826–9834; doi: 10.1021/acs.analchem.2c01670 35759403

[41] Fleischhacker M, Schmidt B. Circulating nucleic acids (CNAs) and cancer—A survey. Biochim Biophys Acta 2007;1775(1):181–232; doi: 10.1016/j.bbcan.2006.10.001 17137717

[42] Ali Z, Aman R, Mahas A, et al. iSCAN: An RT-LAMP-coupled CRISPR-Cas12 module for rapid, sensitive detection of SARS-CoV-2. Virus Res 2020;288:198129; doi: 10.1016/j.virusres.2020.198129 32822689 PMC7434412

[43] Yin K, Ding X, Li Z, et al. Dynamic aqueous multiphase reaction system for one-pot CRISPR-Cas12a-based ultrasensitive and quantitative molecular diagnosis. Anal Chem 2020;92(12):8561–8568; doi: 10.1021/acs.analchem.0c01459 32390420 PMC7588651

[44] Aman R, Marsic T, Sivakrishna Rao G, et al. iSCAN-V2: A one-pot RT-RPA-CRISPR/Cas12b assay for point-of-care SARS-CoV-2 detection. Front Bioeng Biotechnol 2021;9:800104; doi: 10.3389/fbioe.2021.800104 35127671 PMC8815761

[45] Guyard A, Boyez A, Pujals A, et al. DNA degrades during storage in formalin-fixed and paraffin-embedded tissue blocks. Virchows Arch 2017;471(4):491–500; doi: 10.1007/s00428-017-2213-0 28812131

[46] Watanabe M, Hashida S, Yamamoto H, et al. Estimation of age-related DNA degradation from formalin-fixed and paraffin-embedded tissue according to the extraction methods. Exp Ther Med 2017;14(3):2683–2688; doi: 10.3892/etm.2017.4797 28962212 PMC5609301

