# Supplementary material for: In Vitro CRISPR-Cas12a-Based Detection of Cancer-Associated TP53 Hotspot Mutations Beyond the crRNA Seed Region
Source: CRISPR J. 2023 Apr 13;6(2):127–39. doi: 10.1089/crispr.2022.0077 (PMC10123810; doi:10.1089/crispr.2022.0077)
Supplement: Supplemental data [file Suppl_TableS1.docx]

**Table S1. Overview of used oligonucleotides.**

| **Oligo no**. | **Name** | **Sequence (5'-->3')** | **Used for** |
| --- | --- | --- | --- |
| KD001 | TP53_R273wt_fw | AATTCACGGAACAGCTTTGAGGTG**CGT**GTTTGTGCCTGTCCTGGGGGTAC | Annealing with complementary oligo to form short 50bp activators with relevant *TP53* mutations in the middle |
| KD002 | TP53_R273wt_rv | CCCCAGGACAGGCACAAAC**ACG**CACCTCAAAGCTGTTCCGTG |  |
| KD003 | TP53_R273C_fw | AATTCACGGAACAGCTTTGAGGTG**TGT**GTTTGTGCCTGTCCTGGGGGTAC |  |
| KD004 | TP53_R273C_rv | CCCCAGGACAGGCACAAAC**ACA**CACCTCAAAGCTGTTCCGTG |  |
| KD005 | TP53_R273H_fw | AATTCACGGAACAGCTTTGAGGTG**CAT**GTTTGTGCCTGTCCTGGGGGTAC |  |
| KD006 | TP53_R273H_rv | CCCCAGGACAGGCACAAAC**ATG**CACCTCAAAGCTGTTCCGTG |  |
| KD007 | TP53_R273L_fw | AATTCACGGAACAGCTTTGAGGTG**CTT**GTTTGTGCCTGTCCTGGGGGTAC |  |
| KD008 | TP53_R273L_rv | CCCCAGGACAGGCACAAAC**AAG**CACCTCAAAGCTGTTCCGTG |  |
| KD009 | TP53_R273S_fw | AATTCACGGAACAGCTTTGAGGTG**AGT**GTTTGTGCCTGTCCTGGGGGTAC |  |
| KD010 | TP53_R273S_rv | CCCCAGGACAGGCACAAAC**ACT**CACCTCAAAGCTGTTCCGTG |  |
| KD011 | TP53_R273G_fw | AATTCACGGAACAGCTTTGAGGTG**GGT**GTTTGTGCCTGTCCTGGGGGTAC |  |
| KD012 | TP53_R273G_rv | CCCCAGGACAGGCACAAAC**ACC**CACCTCAAAGCTGTTCCGTG |  |
| KD013 | TP53_R273P_fw | AATTCACGGAACAGCTTTGAGGTG**CCT**GTTTGTGCCTGTCCTGGGGGTAC |  |
| KD014 | TP53_R273P_rv | CCCCAGGACAGGCACAAAC**AGG**CACCTCAAAGCTGTTCCGTG |  |
|  | | | |
| KR015 | P53 R273wt-AsCas12a crRNA | /AltR1/rUrArArUrUrUrCrUrArCrUrCrUrUrGrUrArGrArUrArGrGrUrGrCrGrUrGrUrUrUrGrUrGrCrCrUrGrUrC/AltR2/ | AsCas12a-compatible crRNAs for detecting the *TP53* p.R273 mutation panel |
| KR016 | P53 R273C-AsCas12a crRNA | /AltR1/rUrArArUrUrUrCrUrArCrUrCrUrUrGrUrArGrArUrArGrGrUrGrUrGrUrGrUrUrUrGrUrGrCrCrUrGrUrC/AltR2/ |  |
| KR017 | P53 R273H-AsCas12a crRNA | /AltR1/rUrArArUrUrUrCrUrArCrUrCrUrUrGrUrArGrArUrArGrGrUrGrCrArUrGrUrUrUrGrUrGrCrCrUrGrUrC/AltR2/ |  |
| KR018 | P53 R273L-AsCas12a crRNA | /AltR1/rUrArArUrUrUrCrUrArCrUrCrUrUrGrUrArGrArUrArGrGrUrGrCrUrUrGrUrUrUrGrUrGrCrCrUrGrUrC/AltR2/ |  |
| KR019 | P53 R273S-AsCas12a crRNA | /AltR1/rUrArArUrUrUrCrUrArCrUrCrUrUrGrUrArGrArUrArGrGrUrGrArGrUrGrUrUrUrGrUrGrCrCrUrGrUrC/AltR2/ |  |
| KR020 | P53 R273G-AsCas12a crRNA | /AltR1/rUrArArUrUrUrCrUrArCrUrCrUrUrGrUrArGrArUrArGrGrUrGrGrGrUrGrUrUrUrGrUrGrCrCrUrGrUrC/AltR2/ |  |
| KR021 | P53 R273P-AsCas12a crRNA | /AltR1/rUrArArUrUrUrCrUrArCrUrCrUrUrGrUrArGrArUrArGrGrUrGrCrCrUrGrUrUrUrGrUrGrCrCrUrGrUrC/AltR2/ |  |
| KR022 | P53 R273wt-LbCas12a crRNA | /AltR1/rUrArArUrUrUrCrUrArCrUrArArGrUrGrUrArGrArUrArGrGrUrGrCrGrUrGrUrUrUrGrUrGrCrCrUrGrUrC/AltR2/ | LbCas12a-compatible crRNAs for detecting the *TP53* p.R273 mutation panel |
| KR023 | P53 R273C-LbCas12a crRNA | /AltR1/rUrArArUrUrUrCrUrArCrUrArArGrUrGrUrArGrArUrArGrGrUrGrUrGrUrGrUrUrUrGrUrGrCrCrUrGrUrC/AltR2/ |  |
| KR024 | P53 R273H-LbCas12a crRNA | /AltR1/rUrArArUrUrUrCrUrArCrUrArArGrUrGrUrArGrArUrArGrGrUrGrCrArUrGrUrUrUrGrUrGrCrCrUrGrUrC/AltR2/ |  |
| KR025 | P53 R273L-LbCas12a crRNA | /AltR1/rUrArArUrUrUrCrUrArCrUrArArGrUrGrUrArGrArUrArGrGrUrGrCrUrUrGrUrUrUrGrUrGrCrCrUrGrUrC/AltR2/ |  |
| KR026 | P53 R273S-LbCas12a crRNA | /AltR1/rUrArArUrUrUrCrUrArCrUrArArGrUrGrUrArGrArUrArGrGrUrGrArGrUrGrUrUrUrGrUrGrCrCrUrGrUrC/AltR2/ |  |
| KR027 | P53 R273G-LbCas12a crRNA | /AltR1/rUrArArUrUrUrCrUrArCrUrArArGrUrGrUrArGrArUrArGrGrUrGrGrGrUrGrUrUrUrGrUrGrCrCrUrGrUrC/AltR2/ |  |
| KR028 | P53 R273P-LbCas12a crRNA | /AltR1/rUrArArUrUrUrCrUrArCrUrArArGrUrGrUrArGrArUrArGrGrUrGrCrCrUrGrUrUrUrGrUrGrCrCrUrGrUrC/AltR2/ |  |
| KR029 | neg ctrl-AsCas12a crRNA#1 | /AltR1/rUrArArUrUrUrCrUrArCrUrCrUrUrGrUrArGrArUrCrGrUrUrArArUrCrGrCrGrUrArUrArArUrArCrGrG/AltR2/ | crRNAs targeting a synthetic sequence not found in the human genome |
| KR030 | neg ctrl-LbCas12a crRNA#1 | /AltR1/rUrArArUrUrUrCrUrArCrUrArArGrUrGrUrArGrArUrCrGrUrUrArArUrCrGrCrGrUrArUrArArUrArCrGrG/AltR2/ |  |
|  |  |  |  |
| KR038 | Lbcr R273wt PAM+8 U>C | /AltR1/rUrArArUrUrUrCrUrArCrUrArArGrUrGrUrArGrArUrArGrGrUrGrCrGrCrGrUrUrUrGrUrGrCrCrUrGrUrC/AltR2/ | Double mismatch crRNAs |
| KR039 | Lbcr R273 PAM+9 G>U | /AltR1/rUrArArUrUrUrCrUrArCrUrArArGrUrGrUrArGrArUrArGrGrUrGrCrGrUrUrUrUrUrGrUrGrCrCrUrGrUrC/AltR2/ |  |
| KR040 | Lbcr R273 PAM+10 U>C | /AltR1/rUrArArUrUrUrCrUrArCrUrArArGrUrGrUrArGrArUrArGrGrUrGrCrGrUrGrCrUrUrGrUrGrCrCrUrGrUrC/AltR2/ |  |
| KR041 | Lbcr R273 PAM+11 U>C | /AltR1/rUrArArUrUrUrCrUrArCrUrArArGrUrGrUrArGrArUrArGrGrUrGrCrGrUrGrUrCrUrGrUrGrCrCrUrGrUrC/AltR2/ |  |
| KR042 | Lbcr R273 PAM+12 U>C | /AltR1/rUrArArUrUrUrCrUrArCrUrArArGrUrGrUrArGrArUrArGrGrUrGrCrGrUrGrUrUrCrGrUrGrCrCrUrGrUrC/AltR2/ |  |
| KR043 | Lbcr R273 PAM+13 G>C | /AltR1/rUrArArUrUrUrCrUrArCrUrArArGrUrGrUrArGrArUrArGrGrUrGrCrGrUrGrUrUrUrCrUrGrCrCrUrGrUrC/AltR2/ |  |
| KR044 | Lbcr R273 PAM+14 U>C | /AltR1/rUrArArUrUrUrCrUrArCrUrArArGrUrGrUrArGrArUrArGrGrUrGrCrGrUrGrUrUrUrGrCrGrCrCrUrGrUrC/AltR2/ |  |
| KR045 | Lbcr R273 PAM+15 G>C | /AltR1/rUrArArUrUrUrCrUrArCrUrArArGrUrGrUrArGrArUrArGrGrUrGrCrGrUrGrUrUrUrGrUrCrCrCrUrGrUrC/AltR2/ |  |
|  | | | |
| KD035 | FAM-IABkFQ reporter | 6FAM-TTATT-3IABkFQ | FQ-probe |
|  | | | |
| KD046 | R273 PCR_fw | AGTGGTAATCTACTGGGACGGA | Primer pair used for PCR |
| KD047 | R273 PCR_rv | CTGTGCGCCGGTCTCTC |  |
| KD048 | R273 M13 tail_fw | TGTAAAACGACGGCCAGTAGTGGTAATCTACTGGGACGGA | Primer pair used for PCR prior to Sanger sequencing |
| KD049 | R273 M13 tail_rv | CAGGAAACAGCTATGACCTGTGCGCCGGTCTCTC |  |
|  | | | |
| KD050 | M13_fw | TGTAAAACGACGGCCAGT | M13 sequencing primers |
| KD051 | M13_rv | CAGGAAACAGCTATGAC |  |
