# Supplementary figures and images for: In Vitro CRISPR-Cas12a-Based Detection of Cancer-Associated TP53 Hotspot Mutations Beyond the crRNA Seed Region
Source: CRISPR J. 2023 Apr 13;6(2):127–39. doi: 10.1089/crispr.2022.0077 (PMC10123810; doi:10.1089/crispr.2022.0077)

**Supplementary figure S1. Cas12a *in vitro* *cis*-cleavage analysis on supercoiled plasmids.**


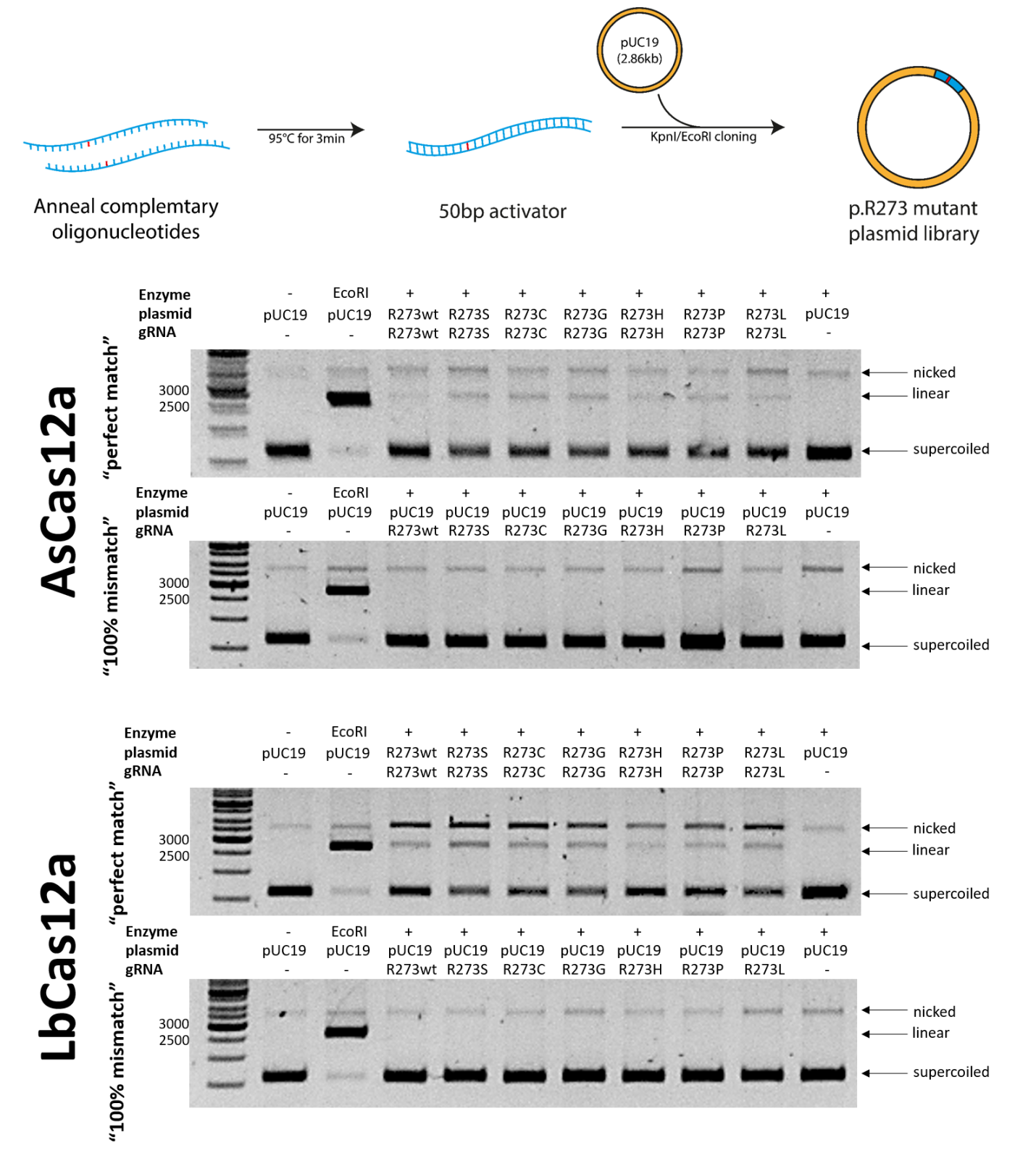

Supplement: Supplemental data [file Suppl_FigS1.docx]
