# Supplementary material for: In Vitro CRISPR-Cas12a-Based Detection of Cancer-Associated TP53 Hotspot Mutations Beyond the crRNA Seed Region
Source: CRISPR J. 2023 Apr 13;6(2):127–39. doi: 10.1089/crispr.2022.0077 (PMC10123810; doi:10.1089/crispr.2022.0077)

**Supplementary figure S2. Combinatorial calibration assays using the LightCycler480 qPCR thermocycler.** (A) Overview of the presence [1] or absence [0] of reagents in reactions C1-C16 of the combinatorial assay. (B) Mean fluorescence values of 3 replicate combinatorial assays, plotted aginst time, measured on a LightCycler480 qPCR thermocycler. C16 is the only reaction yielding significant fluorescence above background. (C) Mean curve slopes from 3 replicate combinatorial assays measured on a LightCycler480 qPCR thermocycler. Error bars display standard error of the mean.


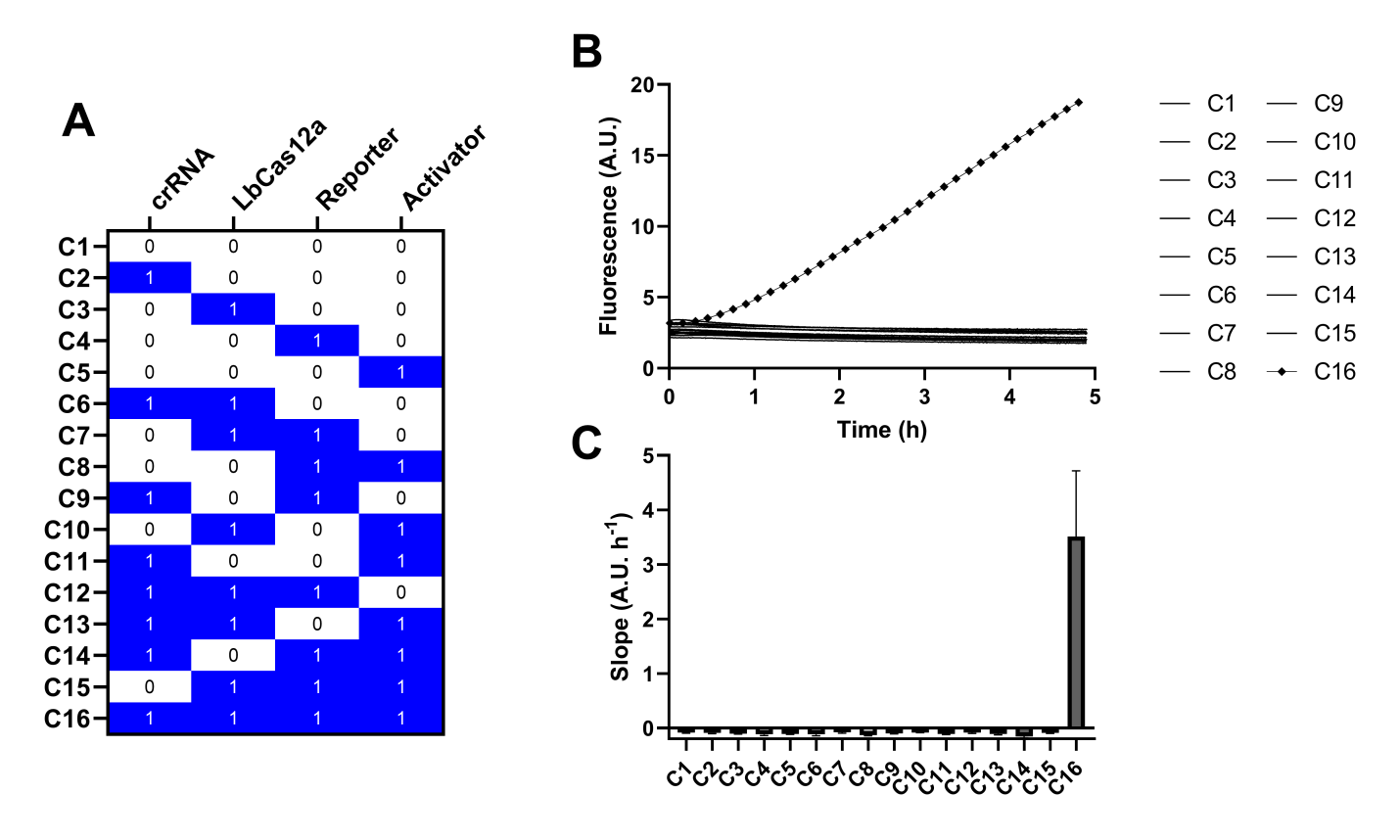

Supplement: Supplemental data [file Suppl_FigS2.docx]
