# Supplementary material for: In Vitro CRISPR-Cas12a-Based Detection of Cancer-Associated TP53 Hotspot Mutations Beyond the crRNA Seed Region
Source: CRISPR J. 2023 Apr 13;6(2):127–39. doi: 10.1089/crispr.2022.0077 (PMC10123810; doi:10.1089/crispr.2022.0077)
Supplement: Supplemental data [file Suppl_FigS3.docx]

**Supplementary figure S3. Comparing the activity of engineered versus wild type Cas12a.** Collateral cleavage activity of different concentrations (A) LbCas12a; (B) LbCas12a Ultra; (C) AsCas12a V3 and (D) AsCas12a Ultra show different enzyme reaction concentrations yield different reaction kinetics. (E) Similar time-dependent activity was found when using 20nM LbCas12a and 4nM of the other enzymes. Graphs represent mean fluorescence values from 3 replicate reactions, measured on a Tecan infinite 200 pro plate reader.
