# Supplementary material for: In Vitro CRISPR-Cas12a-Based Detection of Cancer-Associated TP53 Hotspot Mutations Beyond the crRNA Seed Region
Source: CRISPR J. 2023 Apr 13;6(2):127–39. doi: 10.1089/crispr.2022.0077 (PMC10123810; doi:10.1089/crispr.2022.0077)
Supplement: Supplemental data [file Suppl_FigS5.docx]

**Supplementary figure S5. Raw mismatch analysis data for AsCas12a V3 and AsCas12a Ultra.** (A) Mean background subtracted fluorescence data obtained using 4 nM AsCas12a V3. (B) Curve slopes calculated from the graphs in [A]. (C) Mean background subtracted fluorescence data obtained using 4 nM AsCas12a Ultra. (D) Curve slopes calculated from the graphs in [C]. Graphs represent triplicate experiments, bars display mean slope values (N=3), and are grouped per crRNA.
