# Supplementary material for: In Vitro CRISPR-Cas12a-Based Detection of Cancer-Associated TP53 Hotspot Mutations Beyond the crRNA Seed Region
Source: CRISPR J. 2023 Apr 13;6(2):127–39. doi: 10.1089/crispr.2022.0077 (PMC10123810; doi:10.1089/crispr.2022.0077)
Supplement: Supplemental data [file Suppl_FigS6.docx]

**Supplementary figure S6. Influences of concentration and mismatches.** (A) Increasing the Cas12a concentrations has minimal effect on the specificity profiles as observed in figure 3. Curve slopes were calculated from background-subtracted data and were percentually normalized to the steepest slope value for a given activator. Heat maps represent three independently performed experiments. Heat maps for 20 nM LbCas12a and 4 nM LbCas12a Ultra are the same as in figure 3. (B) The designed double mismatch crRNAs are p.R273wt-detecting crRNAs that contain an additional mismatch that would disfavor the off-target effect on p.R273H activator. (C) Normalized slopes in the displayed heat map identified promising candidates with strongly reduced off-target effect, nevertheless (D) the intended activity for wild type detection was at least reduced by half.
