# Supplementary material for: In Vitro CRISPR-Cas12a-Based Detection of Cancer-Associated TP53 Hotspot Mutations Beyond the crRNA Seed Region
Source: CRISPR J. 2023 Apr 13;6(2):127–39. doi: 10.1089/crispr.2022.0077 (PMC10123810; doi:10.1089/crispr.2022.0077)
Supplement: Supplemental data [file Suppl_FigS8.docx]

**Supplementary figure S8. Fluorescence signal of wild type LbCas12a-based detection of mutant p.R273 codons in patient biopsies.** Curves represent background-subtracted mean fluorescence values of triplicate reactions, error bars display standard error of the mean.
