# Supplementary material for: In Vitro CRISPR-Cas12a-Based Detection of Cancer-Associated TP53 Hotspot Mutations Beyond the crRNA Seed Region
Source: CRISPR J. 2023 Apr 13;6(2):127–39. doi: 10.1089/crispr.2022.0077 (PMC10123810; doi:10.1089/crispr.2022.0077)
Supplement: Supplemental data [file Suppl_FigS9.docx]

**Supplementary figure S9. Sequencing data of the tumor biopsies of 2 HNSCC patients.** *TP53* p.R273 Sanger sequencing chromatogram data show presence of tumor-specific p.R273H alleles in patient 2. DNA was purified from macrodissected tissue with at least 20% neoplastic cellularity. Tissue heterozygosity and presence of non-neoplastic cells in the tumor biopsy are a likely source for the guanine peak representing wild type *TP53* alleles.
